# Fermentative *N*-Methylanthranilate Production by Engineered *Corynebacterium glutamicum*

**DOI:** 10.3390/microorganisms8060866

**Published:** 2020-06-08

**Authors:** Tatjana Walter, Nour Al Medani, Arthur Burgardt, Katarina Cankar, Lenny Ferrer, Anastasia Kerbs, Jin-Ho Lee, Melanie Mindt, Joe Max Risse, Volker F. Wendisch

**Affiliations:** 1 Genetics of Prokaryotes, Faculty of Biology and CeBiTec, Bielefeld University, 33615 Bielefeld, Germany; t.walter@uni-bielefeld.de (T.W.); mohamad.al@uni-bielefeld.de (N.A.M.); arthur.burgardt@uni-bielefeld.de (A.B.); lferrer@cebitec.uni-bielefeld.de (L.F.); anastasia.kerbs@uni-bielefeld.de (A.K.); melanie.mindt@wur.nl (M.M.); 2 BU Bioscience, Wageningen University & Research, 6700AA Wageningen, The Netherlands; katarina.cankar@wur.nl; 3 Major in Food Science & Biotechnology, School of Food Biotechnology & Nutrition, Kyungsung University, Busan 48434, Korea; jhlee83@ks.ac.kr; 4 Fermentation Technology, Technical Faculty and CeBiTec, Bielefeld University, 33615 Bielefeld, Germany; jrisse@uni-bielefeld.de

**Keywords:** *N*-functionalized amines, *N*-methylanthranilate, *Corynebacterium glutamicum*, metabolic engineering, sustainable production of quinoline precursors, acridone, quinazoline alkaloid drugs

## Abstract

The *N*-functionalized amino acid *N*-methylanthranilate is an important precursor for bioactive compounds such as anticancer acridone alkaloids, the antinociceptive alkaloid *O*-isopropyl *N*-methylanthranilate, the flavor compound *O*-methyl-*N*-methylanthranilate, and as a building block for peptide-based drugs. Current chemical and biocatalytic synthetic routes to *N*-alkylated amino acids are often unprofitable and restricted to low yields or high costs through cofactor regeneration systems. Amino acid fermentation processes using the Gram-positive bacterium *Corynebacterium glutamicum* are operated industrially at the million tons per annum scale. Fermentative processes using *C. glutamicum* for *N*-alkylated amino acids based on an imine reductase have been developed, while *N*-alkylation of the aromatic amino acid anthranilate with *S*-adenosyl methionine as methyl-donor has not been described for this bacterium. After metabolic engineering for enhanced supply of anthranilate by channeling carbon flux into the shikimate pathway, preventing by-product formation and enhancing sugar uptake, heterologous expression of the gene *anmt* encoding anthranilate *N*-methyltransferase from *Ruta graveolens* resulted in production of *N*-methylanthranilate (NMA), which accumulated in the culture medium. Increased SAM regeneration by coexpression of the homologous adenosylhomocysteinase gene *sahH* improved *N*-methylanthranilate production. In a test bioreactor culture, the metabolically engineered *C. glutamicum* C1* strain produced NMA to a final titer of 0.5 g·L^−1^ with a volumetric productivity of 0.01 g·L^−1^·h^−1^ and a yield of 4.8 mg·g^−1^ glucose.

## 1. Introduction

*N*-Functionalization of natural products as well as fine and bulk chemicals includes *N*-hydroxylation, *N*-acetylation, *N*-phosphorylation, or *N*-alkylation. These amine and amino acid modifications are found in all domains of life, and they fulfill various physiological roles such as resistance of bacteria to the antibiotic rifampicin by its *N*-hydroxylation [1], biosynthesis of the hormone melatonin via *N*-acetylated serotonin in plants and mammals [2], or assimilation of methylamine as carbon and energy source in methylotrophic bacteria [3].

The biotechnological and chemical interest in *N*-functionalized amines, especially in *N*-alkylated amino acids, has increased recently because of their beneficial impact as building blocks when incorporated into peptide-based drugs. Better membrane permeability, increased stability against proteases, stabilization of discrete confirmations, prevention of peptide aggregation by reduced formation of hydrogen bonds, or increased receptor subtype selectivity were shown for peptide-based drugs as consequence of amino acid *N*-alkylation [4]. For example, *N*-methylation of the *N*–Cα peptide bonds of transition state mimetics developed to inhibit malarial protease, which is required for infecting erythrocytes, improved their lipophilicity and stability against proteolysis, thus enhancing activity against *Plasmodium* parasites [5]. Free *N*-alkylated amines such as the *N*-ethylated glutamine derivative l-theanine, which prominently occurs in green tea, or *O*-methyl-*N*-methylanthranilate of grapes are flavoring compounds with applications in the food, cosmetics, flavor, and fragrances industries.

Chemical synthesis of free *N*-alkylated amino acids is well studied, and various routes are known, such as by nucleophilic substitution of α-bromo acids, *N*-methylation of sulfonamides, carbamates or amides, reduction of Schiff bases generated with an amino acid and formaldehyde or other aldehydes, by direct alkylation of protected amino acids or by ring-opening of 5-oxazolidinones [6,7,8,9]. However, these processes are often limited by low product yields, over-methylation, toxic reagents, or their incomplete enantiopurity [10,11]. Recently, enzyme catalysis routes with *N*-methyltransferases, dehydrogenases, ketimine reductases, or imine reductases that depend on cofactor regeneration systems have been described [12]. Fermentation processes using simple mineral salts media have been developed for three different routes for de novo production of *N*-alkylated amino acids. Two metabolic engineering strategies for reductive alkylamination of 2-oxo acids with monomethylamine that either make use of a C1-assimilation pathway present in methylotrophic bacteria [13] or of the imine reductase DpkA [14] have been established. *S*-Adenosyl-l-methionine (SAM)-dependent methylation of aromatic amino acids by *N*-methyltransferases has also been described [15].

*N*-methylanthranilate (NMA) is an intermediate of the acridone alkaloid biosynthesis in plants. The SAM-dependent transfer of a methyl group to anthranilate initiates the biosynthesis of NMA-dependent biosynthesis of *N*-methylated acridone alkaloids and avenacin in plants [16,17]. Until now only one *N*-methyltransferase enzyme ANMT was characterized from the common rue, *Ruta graveolens* L., which accumulates *N*-methylated acridones exclusively. This enzyme shows narrow specificity for anthranilate, not accepting methylated catechol, salicylate, caffeate, 3- and 4-hydroxybenzoate, and anthraniloyl-CoA as substrates [16]. The acridone alkaloids and avenacin pathways diverge after SAM-dependent *N*-methylation of acridone anthranilate with regard to activation for transfer to the respective alkaloid intermediate. An ATP-dependent transfer of CoA is postulated for the acridone alkaloid biosynthesis [18], while UDP glucose-dependent *O*-glycosylation was shown as second step of the avenacin biosynthesis [17]. Acridone alkaloids and avenacin are known as bioactive compounds with cytotoxic, anticancer, antimicrobial, or antiparasitic properties and are, therefore, used for pharmaceutical and therapeutic purposes. Several *N*-methylated acridones, namely citrusamine, evoxanthine, arborinine, or normelicopine, were identified in diverse plants [19]. Arborinine, as an example, was found in ethyl acetate extracts from *Glycosmis parva,* and it showed anticancer activity against human cervical cancer cells since activation of caspase-dependent apoptosis without inducing the DNA damage response was observed [20]. *N*-methylanthranilate also serves as precursor for the flavoring agent *O*-methyl-*N*-methylanthranilate, which has an orange blossom and grape-like odor, the antinociceptive alkaloid *O*-isopropyl-*N*-methylanthranilate, or the anti-inflammatory active compound *O*-propyl-*N*-methylanthranilate [21,22,23].

Safe production of amino acids for the food and feed industry has been established at the annual million-ton scale for decades with *Corynebacterium glutamicum* as the dominant production host [24]. *C. glutamicum* grows on simple mineral salts media and can utilize various sugars [25,26], acids such as citrate [27], and alcohols such as ethanol [28]. A well-established toolbox enabled metabolic engineered-based approaches for production of diverse value-added compounds. Besides the production of proteinogenic amino acids, also a broad range of non-proteinogenic amino acid products like γ-aminobutyrate [29], 5-aminovalerate [30,31], pipecolic acid [32,33], *N*-methylated amino acids like *N*-methylalanine (NMeAla) [34] and sarcosine [35], aromatic compounds like 4-hydroxybenzoate [36,37] or protocatechuic acid [38], and functionalized aromatics like 7-chloro- or 7-bromo-tryptophan [39,40] and *O*-methylanthranilate [41] have been demonstrated.

**Figure 1:**
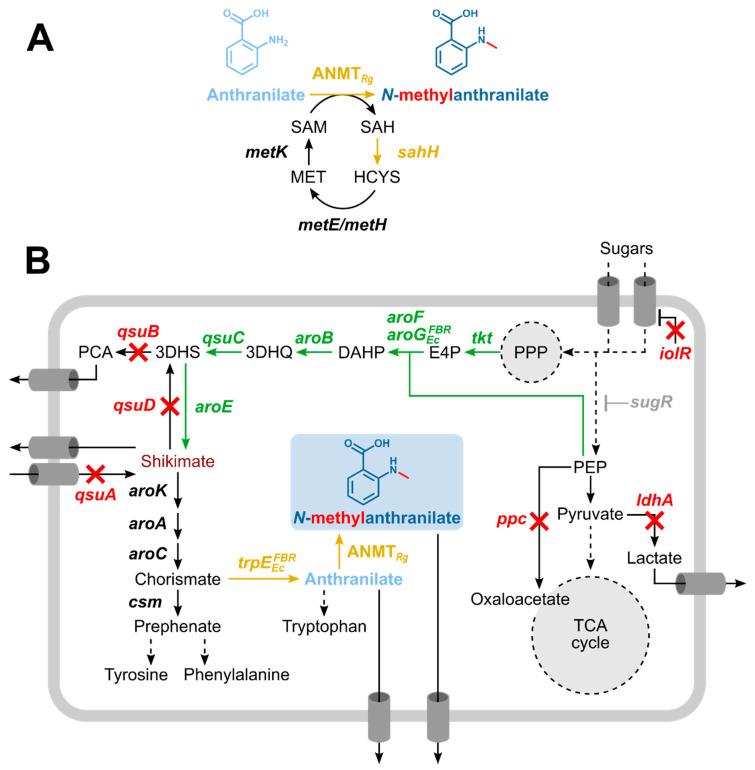
Schematic representation of *N*-methylanthranilate (NMA) biosynthesis (**A**) embedded into aromatic amino acid metabolism of engineered *C. glutamicum* (**B**). Continuous arrows indicate single reactions, dashed arrows indicate multiple reactions. Green arrows and gene names indicate genome-based overexpression, yellow arrows and gene names indicate vector-based expression, crossed arrows and red gene names indicate gene deletion. (**A**) *N*-methylation of anthranilate by *N*-methylanthranilate transferase (ANMT) from *R. graveolens* under consumption of *S*-adenosylmethionine (SAM). The SAM regeneration cycle is depicted with overexpression of *sahH*, S-adenosylhomocysteine hydrolase. SAH, *S*-adenosylhomocysteine; HCYS, l-homocysteine; MET, l-methionine; *metE/metH*, methionine synthase; *metK*, methionine adenosyltransferase. (**B**) Strain engineering towards production of NMA. Grey *sugR* indicates reversion of deleted *sugR* back to wild type *sugR*. PEP, phosphoenolpyruvate; TCA, tricarboxylic acid; PPP, pentose phosphate pathway; E4P, erythrose-4-phosphate; DAHP, 3-deoxy-d-arabinoheptulosonate-7-phosphate; 3DHQ, 3-dehydroquinate; 3DHS, 3-dehydroshikimic acid; PCA, protocatechuic acid; *iolR*, transcriptional regulator; *sugR*, transcriptional regulator; *ppc*, phosphoenolpyruvate carboxylase; *ldhA*, lactate dehydrogenase; *tkt*, transketolase; *aroF*, DAHP synthase; *aroG^FBR^*, feedback-resistant DAHP synthase from *Escherichia coli*; *aroB*, 3-dehydroquinate synthase; *qsuC*, 3-dehydroquinate dehydratase; *qsuB*, 3-dehydroshikimate dehydratase; *qsuD*, shikimate dehydrogenase; *aroE*, shikimate dehydrogenase; *qsuA*, putative shikimate importer; *aroK*, shikimate kinase; *aroA*, 5-enolpyruvylshikimate-3-phosphate synthase; *aroC*, chorismate synthase; *csm*, chorismate mutase; *trpE^FBR^*, feedback-resistant anthranilate synthase from *E. coli*.

Here, we describe fermentative *N*-methylanthranilate production by metabolic engineering of genome-reduced chassis strain *C. glutamicum* C1*, a robust basic strain for synthetic biology and industrial biotechnology [42]. Fermentative NMA production from glucose involved SAM-dependent ANMT from *R. graveloens* combined with metabolic engineering for efficient supply of the precursor anthranilate (Figure 1).

## 2. Materials and Methods

### 2.1. Bacterial Strains and Culture Conditions

All bacterial strains used are listed in Table 1. *Escherichia coli* DH5α [43] was used for plasmid construction. *C. glutamicum* C1* was used as host organism for shikimate, anthranilate, and NMA production. Pre-cultures of *E. coli* and *C. glutamicum* were performed in lysogeny broth (LB) and brain heart infusion (BHI) medium at 37 or 30 °C in baffled shake flasks on a rotary shaker (160 rpm or 120 rpm). Cultures were inoculated freshly from LB agar plates. When necessary, spectinomycin (100 µg·mL^−1^) and kanamycin (25 µg·mL^−1^) were added to the medium. For induction of gene expression from vectors pEKEx3 and pGold, isopropyl-β-d-1-thiogalactopyranoside (IPTG) was added to the medium. For the performance of growth or production experiments of *C. glutamicum*, pre-cultures were inoculated as described above. After cell harvesting (3200× *g*, 7 min), cells were washed with TN-buffer pH 6.3 (50 mM Tris-HCL, 50 mM NaCl) and inoculated to an optical density at 600 nm (OD_600_) of 1 in CGXII minimal medium [44] and 40 g glucose as sole carbon source. *C. glutamicum* grown in 500 mL baffled shake flasks was followed by measuring OD_600_ using a V-1200 spectrophotometer (VWR, Radnor, PA, USA). An OD_600_ of 1 was determined to be equivalent to a biomass concentration of 0.25 g cell dry weight per liter.

Evaluation of the effects of anthranilate and NMA on *C. glutamicum* growth was performed in the microbioreactor system Biolector (m2p-labs; Aachen, Germany). Pre-cultures were grown in BHI-rich medium overnight and transferred to second pre-culture of CGXII minimal medium with 40 g·L^−1^ glucose until the early exponential phase before inoculating to the main medium of CGXII minimal medium and 40 g·L^−1^ glucose with addition of varying anthranilate (solved in water) and NMA (solved in methanol) concentrations. Each condition with NMA contained 1.65 M methanol. Growth experiments in the Biolector were carried out using 48-well flower plates (MTP-48-B; m2p-labs) with a filling volume of 1 mL, at 30 °C, and 1200 rpm shaking frequency. Humidity was kept constant at 85%, and online biomass measurements of scattered light were monitored with backscatter gain of 20.

### 2.2. Fed-Batch Cultivation

Fed-Batch fermentation of *C. glutamicum* NMA105 was performed in an initial volume of 2 L in a bioreactor (3.7 L KLF, Bioengineering AG, 8636 Wald, Switzerland) at 30 °C, 0.2 bar overpressure, and an aeration rate of 2 NL·min^−1^. We did not perform off-gas analysis. To maintain relative dissolved oxygen saturation at 30%, stirrer speed was controlled during growth. The pH was maintained at pH 7.0 due to controlled addition of KOH (4 M) and phosphoric acid (10% (*w*/*w*)). To avoid foaming, the antifoam Sruktol^®^ J647 was added manually when necessary. Feeding with 400 g·L^−1^ glucose and 150 g·L^−1^ (NH_4_)_2_SO_4_ (total volume: 500 mL) was activated when the relative dissolved oxygen saturation (rDOS) signal rose above 60% and stopped when rDOS fell below 60%. Samples were taken automatically every 4 h during the whole cultivation and cooled down to 4 °C until further use. *C. glutamicum* NMA105 cells were transferred from a first pre-culture grown in LB in shake flasks to a second pre-culture in standard CGXII (pH 7.0) medium with 40 g·L^−1^ glucose (without IPTG) and the required antibiotics. For the bioreactor culture, standard CGXII medium without addition of 3-(*N*-morpholino)propanesulfonic acid (MOPS) and antibiotics was used. The fermenter was inoculated with the second pre-culture to an OD of 1.5 and immediately induced with 1 mM of IPTG.

### 2.3. Molecular Genetic Techniques and Strain Construction

Standard molecular genetic techniques were performed as described [46]. Competent *E. coli* DH5α [43] was performed with the RbCl method and transformed by heat shock [46]. Transformation of *C. glutamicum* was performed by electroporation [44]. The gene *trpE*^FBR^ was amplified using specific primers (Table 2) with ALLin^TM^ HiFi DNA Polymerase (highQu GmbH, Kraichtal, Germany). The PCR products were assembled with *Bam*HI restricted pEKEx3 via Gibson Assembly [44].

For heterologous expression of the *N*-methylanthranilate transferase gene, firstly, the pEC-XK99E vector was modified to be suitable for Golden Gate based modular assembly of multiple genes simultaneously. To this end, the three *Bsa*I sites present in the vector located in the *rrnB* terminator, the vector backbone, and the *repA* ORF were removed. Next, a linker containing two *Bsa*I sites (CAGATGAGACCGCATGCCTGCAAGGTCTCAGTAT) was added to the MCS between *Eco*RI and *Sac*I restriction sites. The resulting vector was named pGold (GenBank: MT521917). The coding sequence (CDS) of the plant gene *anmt* (GenBank: DQ884932.1) encoding the *N*-methylanthranilate transferase of *Ruta graveolens* was codon-harmonized to the natural codon frequency of *C. glutamicum* ATCC13032 with the codon usage table of kazusa database [47] and synthesized with Golden Gate assembly compatible flanking regions including recognition site for the restriction enzyme type 2 *B*sa*I* and pGold complementary sequences and an optimized RBS [48,49] (Supplementary Data Table S1). The gene *anmt* was amplified using specific primers (Table 2) with ALLin^TM^ HiFi DNA Polymerase according to the manufacturer (highQu GmbH, Kraichtal, Germany). The PCR products were assembled with digested pGold-*anmt* with *Bam*HI via Gibson Assembly [44].

Chromosomal gene deletions and replacements in C1*-derived strains were performed by two-step homologous recombination [44] using the suicide vector pK19*mobsacB* [50]. The genomic regions flanking the respective gene for homologous recombination were amplified from *C. glutamicum* WT as described elsewhere [51] using the respective Primer pairs containing artificial RBS ([48,49], Table 2). The purified PCR products were assembled and simultaneously cloned into restricted pK19*mobsacB* by Gibson Assembly resulting in the plasmids listed in Table 3. Transfer of the suicide vectors was carried out by trans-conjugation using *E. coli* S17 as donor strain [33]. For the first recombination event, integration of the vector in one of the targeted flanking regions was selected via kanamycin resistance. The resulting clones showed sucrose sensitivity due to the levansucrase gene *sacB*. Suicide vector excision was selected by sucrose resistance. Gene deletions or replacements were verified by PCR and sequencing with respective primers (Table 2).

### 2.4. Quantification of Amino Acids and Organic Acids

Extracellular amino acids and carbohydrates were quantified by high-performance liquid chromatography (HPLC) (1200 series, Agilent Technologies Deutschland GmbH, Böblingen, Germany). The culture supernatants were collected at different time points and centrifuged (20,200× *g*) for HPLC analysis.

For the detection of α-ketoglutarate (α-KG), trehalose, and lactate, an amino exchange column (Aminex, 300 mm × 8 mm, 10 µm particle size, 25 Å pore diameter, CS Chromatographie Service GmbH, 52379 Langerwehe, Germany) was used. The measurements were performed under isocratic conditions for 17 min at 60 °C with 5 mM sulfuric acid and a flow rate of 0.8 mL·min ^−1^. The detection was carried out with a Diode Array Detector (DAD, 1200 series, Agilent Technologies, Santa Clara, CA 95051, USA) at 210 nm.

Separation of shikimate, anthranilate, and NMA was performed with a pre-column (LiChrospher 100 RP18 EC-5µ (40 × 4 mm), CS Chromatographie Service GmbH, Langerwehe, Germany) and a main column (LiChrospher 100 RP18 EC-5µ (125 × 4 mm), CS Chromatographie Service GmbH). A mobile phase of buffer A (0.1% trifluoroacetic acid dissolved in water) and buffer B (acetonitrile) was used with a flow rate of 1 mL·min^−1^. The following gradient was applied: 0–1 min 10% B; 1–10 min a linear gradient of B from 10% to 70%; 10–12 min 70% B; 12–14 min a linear gradient of B from 70% to 10%; 14–18 min 10% B [41]. The injection volume was 20 µL, and detection was performed with DAD at 210, 280, and 330 nm.

## 3. Results

### 3.1. Corynebacterium glutamicum as Suitable Host for NMA Production

**Figure 2:**
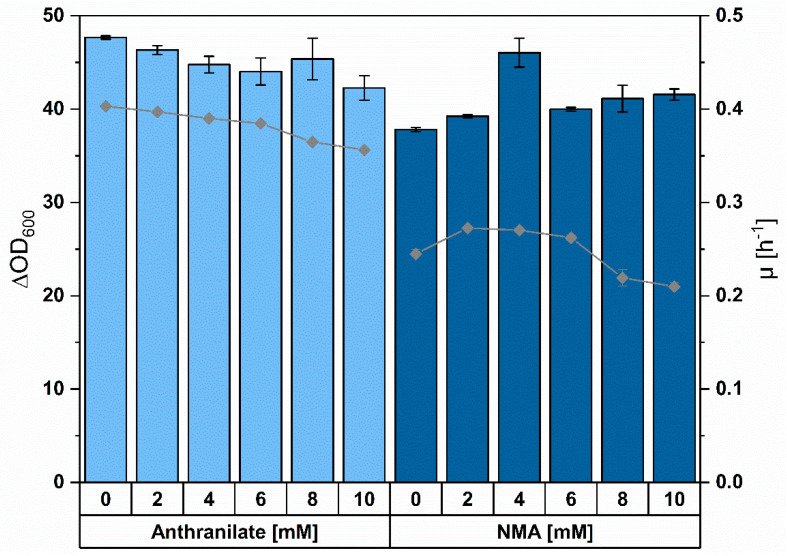
Effect of externally added NMA (bright blue) and anthranilate (dark blue) on biomass formation (columns) and specific growth rate (lines) of *C. glutamicum* strain ATCC13032. Each condition with NMA contained the same amount of methanol (1.65 M) in minimal media. Averages and standard deviation of triplicate cultivations are shown.

*C. glutamicum* is widely used in amino acid fermentation, which operates at a million tons per annum scale [56]; however, it has not been engineered so far for NMA production. As expected, inspection of the genome revealed that there was no gene(s) encoding for a native enzyme that may *N*-methylate anthranilate to yield NMA. To study the growth responses of *C. glutamicum* to anthranilate and NMA, the wild-type strain ATCC13032 (WT) was cultivated with addition of varying anthranilate and NMA concentrations to CGXII minimal medium and 40 g·L^−1^ glucose. Neither anthranilate nor NMA were utilized or converted by *C. glutamicum* WT, since their concentrations in supernatants analyzed at the beginning and the end of cultivation were comparable. Maximal biomass concentrations (expressed as ΔOD_600_) were hardly affected by addition of anthranilate or NMA. By extrapolation, the concentrations of anthranilate (about 36 mM) and NMA (about 34 mM), which reduced the specific growth rate in glucose minimal medium to half-maximal, were determined (Figure 2). Based on the observed tolerance, *C. glutamicum* is a suitable candidate for production of anthranilate and NMA.

### 3.2. Construction of a C. glutamicum Platform Strain for Production of Anthranilate

Since anthranilate, an intermediate of the tryptophan branch in the shikimate pathway, is a direct precursor of NMA, *C. glutamicum* C1* was engineered for increased supply of shikimate pathway intermediates by eliminating bottlenecks and minimizing formation of by-products (Figure 1). Hence, in sequential steps, *aroG*^D146^ encoding feedback resistant 3-deoxy-d-arabino-heptulosonate-7-phosphate (DAHP) synthase from *E. coli* [57] was inserted into the locus of *vdh* coding for vanillin dehydrogenase, which oxidizes vanillin and other aromatic aldehydes such as protocatechic aldehyde [58]. Next, an in-frame deletion of *ldhA* to reduce l-lactate formation (ARO02) and an *sugR* deletion to increase glycolytic gene expression and sugar uptake [59] were introduced to yield strain ARO03.

**Figure 3:**
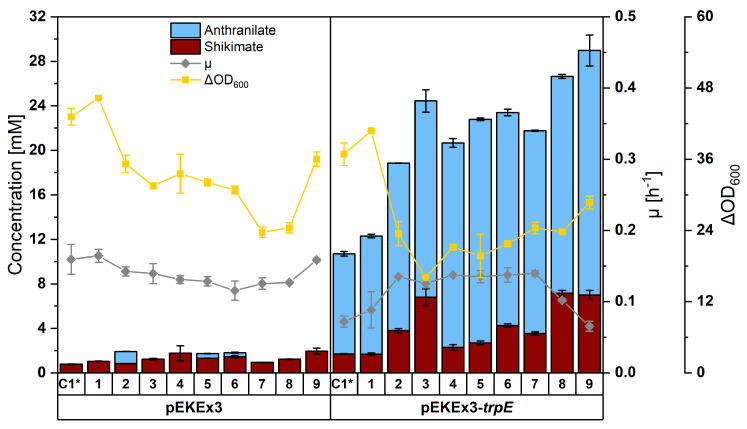
Production of shikimate (maroon bars) and anthranilate (light blue bars), maximal specific growth rate (gray diamonds) and biomass formation (yellow squares) by *C. glutamicum* strains C1* and ARO01 to ARO09 carrying either pEKEx3 (left panel) or pEKEx3-*trpE*^FBR^ (right panel) were grown in shake flasks in CGXII minimal medium with 40 g·L^−1^ glucose for 48 h. Means and arithmetic errors of duplicate cultures are shown.

Upon transformation with pEKEx3 as an empty vector control and pEKEx3-*trpE*^FBR^ for expression of feedback-resistant anthranilate synthase from *E. coli* [60], strains were evaluated regarding their growth behavior, anthranilate production, and formation of by-products. After 48 h of shake flask cultivation, ARO03(pEKEx3) exhibited decreased biomass formation and increased trehalose and α-ketoglutarate accumulation as compared to ARO01(pEKEx3). Expression of *trpE*^FBR^ further decreased biomass formation (i.e., 16.4% less than in empty vector). Comparing strains C1* to ARO03 carrying pEKEx3-*trpE*^FBR^ revealed a stepwise increase both in anthranilate and in shikimate production (Figure 3). For example, ARO03 strain harboring pEKEx3-*trpE*^FBR^ produced 17.6 ± 1.0 mM anthranilate and 6.8 ± 0.8 mM shikimate as compared to C1*(pEKEx3-*trpE*^FBR^) that accumulated only 9.0 ± 0.2 mM anthranilate and 1.7 ± 0.1 mM shikimate.

To further increase the carbon flux towards shikimate, several further metabolic engineering steps were undertaken. In ARO04, the gene *aroR*, which codes for a translational regulatory leader peptide and is located upstream of DHAP synthase gene *aroF* [61], was replaced by an *ilvC* promoter followed by an optimized RBS in order to relieve negative translational control of *aroF* by phenylalanine and tyrosine. As described previously [36], the *qsuABCD* operon was replaced by *qsuC* transcribed from the constitutive strong *tuf* promoter in strain ARO05. This blocked conversion of 3-dehydroshikimate (3-DHS) to the unwanted by-product protocatechuate (PCA) on the one hand and increased the flux from 3-dehydroquinate (3-DHQ) to 3-DHS on the other hand. The replacement of *ppc* encoding phosphoenolpyruvate (PEP) carboxylase by a second copy of endogenous *aroB* encoding 3-DHQ synthase in ARO06 probably increased supply of PEP as precursor for the shikimate pathway, and overexpression of *aroB* increased conversion of DHAP to 3-DHQ. To increase supply of erythrose-4-phosphate (E4P) as second precursor of the shikimate pathway [62], the native promoter upstream of transketolase gene *tkt* was exchanged by the constitutive strong promoter P*tuf* with an artificial RBS. Since *tkt* is co-transcribed with other genes of the pentose phosphate pathway as operon *tkt-tal-zwf-opcA-pgl*, this promoter exchange is expected to increase flux into the pentose phosphate pathway towards E4P in strain ARO07.

**Figure 4:**
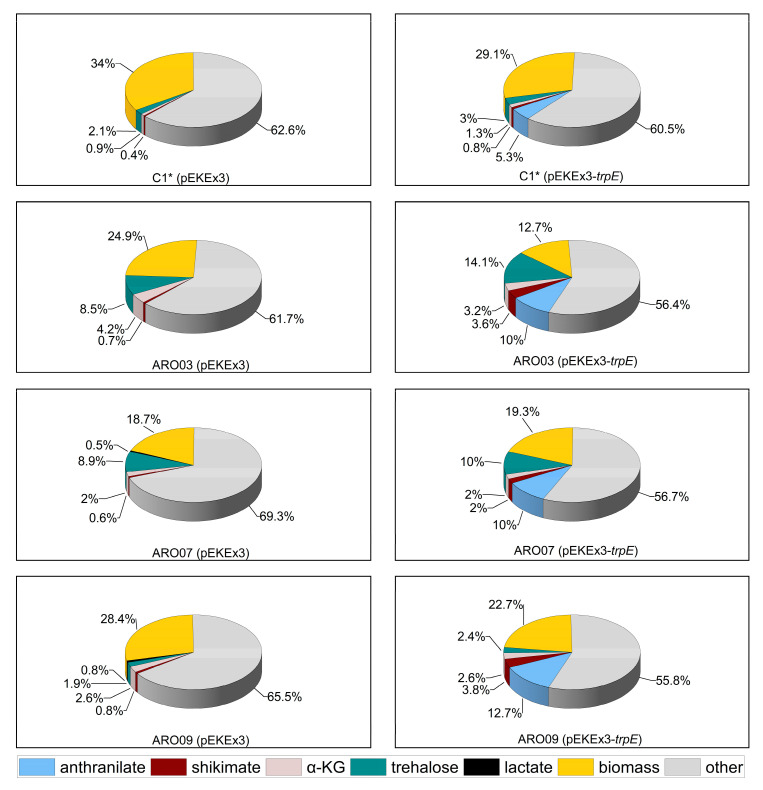
Fate of carbon from glucose in cultivations of *C. glutamicum* ARO strains carrying the empty vector (left) or pEKEx3-*trpE^FBR^* (right). Carbon (given in mol%) derived from glucose found after 48 h in secreted products anthranilate (blue), shikimate (maroon), α-ketoglutarate (light red), trehalose (green), lactate (black) as well as in the formed biomass (yellow) are shown for *C. glutamicum* strains C1*, ARO03, ARO07, and ARO09 harboring either pEKEx3 (left) or pEKEx3-*trpE^FBRfbr^* (right). Carbon that could not be accounted for is depicted in gray (other). Values were determined from duplicate cultures. Experimental error was less than 20%. Abbreviations used: α-KG, α-ketoglutarate. Carbon distribution of all ARO strains can be found in the Supplementary Data (Figure S1; Figure S2).

Upon transformation with pEKEx3-*trpE*^FBR^, ARO07 produced only slightly more anthranilate (18.2 ± 0.1 mM) than ARO03(pEKEx3-*trpE*^FBR^), but less shikimate, trehalose, and α-ketoglutarate (Figure 4). Growth was comparably fast (µ of 0.14 ± 0.01 h^−1^ compared to 0.13 ± 0.01 h^−1^), but a higher biomass was reached (OD_600_ of 24.4 ± 1.0 compared with 16.1 ± 0.1) (Figure 3).

In ARO08, shikimate dehydrogenase gene *aroE* was overexpressed from the strong constitutive promoter P*tuf* and used to replace *iolR.* In the absence of IolR, the inositol catabolism operon (cg0197-cg0207), *cg1268*, and PEP carboxykinase gene *pck* are deregulated [63,64], and *iolT1*, which codes for a non-phosphoenolpyruvate dependent phosphotransferase transporter (non-PTS) inositol uptake system, is derepressed. Non-PTS uptake of glucose is known to improve availability of PEP. The final strain, ARO09, is a *sugR-*positive derivative of ARO08. ARO09(pEKEx3-*trpE*^FBR^) grew faster than ARO7(pEKEx3-*trpE*^FBR^) (Figure 3) and accumulated less trehalose as unwanted by-product. The maximum anthranilate titer of 22.0 ± 1.4 mM (equivalent to about 3.1 g·L^−1^ anthranilate) was achieved with ARO09(pEKEx3-*trpE*^FBR^) after 48 h of shake flask cultivation. This titer was 2.5 times more than that obtained with C1*(pEKEx3-*trpE*^FBR^). Taken together, an anthranilate producing *C. glutamicum* strain converting 12.7% of carbon from glucose (Figure 4) to about 3.1 g·L^−1^ of anthranilic acid, the direct precursor for NMA, was constructed.

### 3.3. Establishing Fermentative Production of NMA by C. glutamicum

NMA is synthesized from anthranilate in a single SAM-dependent methylation reaction at its amino group (Figure 1). Therefore, the anthranilate producing *C. glutamicum* strain ARO09(pEKEx3-*trpE*^FBR^) was used for heterologous expression of the anthranilate *N*-methyltransferase gene *anmt* from *R. graveolens*. Transformation of ARO09(pEKEx3-*trpE*^FBR^) with pGold-*anmt* yielded strain NMA104. To improve SAM regeneration, the endogenous *S*-adenosylhomocysteinase gene *sahH* was expressed as synthetic operon with *anmt* from plasmid pGold-*anmt*-*sahH* and used to transform ARO09(pEKEx3-*trpE*^FBR^) yielding strain NMA105. As negative control, pGold was introduced into ARO09(pEKEx3-*trpE*^FBR^) yielding strain NMA103 (Table 1). For comparison, the shikimate producing strain ARO9(pEKEx3) was transformed with pGold, pGold-*anmt,* and pGold-* anmt*-*sahH* yielding strains NMA100, NMA101, and NMA102, respectively (Table 1).

**Figure 5:**
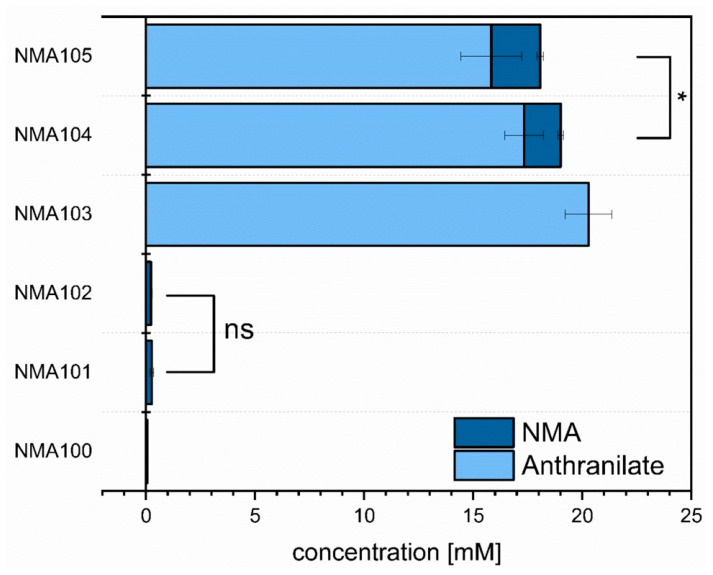
Production of anthranilate (light blue) and NMA (dark blue) by *C. glutamicum* strains NMA100 to NMA105. Cultivation was performed in minimal medium supplemented with 40 g·L^−1^ glucose as carbon source. 1 mM IPTG was added for induction of gene expression. Means and standard deviations of triplicate cultures determined after 48 h cultivation are depicted. Significance has been determined for NMA concentrations based on a two-sided unpaired Student’s t-test (*: *p* < 0.05; ns: not significant).

In order to test for NMA production, strains NMA100 to NMA105 were cultivated in CGXII minimal medium supplemented with 40 g·L^−1^ glucose as carbon source. HPLC analysis of supernatants after cultivation for 48 h revealed that NMA100 and NMA103 did not produce NMA, which was expected since they lacked *anmt* from *R. graveolens* (Figure 5). Expression of *anmt* alone or in combination with endogenous *sahH* resulted in production of about 0.5 mM NMA by strains NMA101 and NMA102, respectively. This indicated functional expression of *anmt* from *R. graveolens* in *C. glutamicum.*

Coexpression of *trpE^FBR^* to boost anthranilate production with *anmt* alone (strain NMA104) resulted in production of 1.7 ± 0.1 mM (0.25 ± 0.02 g·L^−1^) NMA. The finding that the anthranilate concentration was reduced from 20.8 ± 0.0 mM as obtained with NMA103 to 17.3 ± 0.9 mM (NMA104) indicated that conversion of anthranilate to NMA was incomplete (at about 10 mol%). Upon coexpression of *trpE^FBR^* with both *anmt* and *sahH* in strain NMA105, 15.8 ± 1.9 mM anthranilate remained as unconverted precursor (Figure 5), and a significantly increased NMA titer of 2.2 ± 0.2 mM was obtained. This maximal titer in shake flasks corresponds to 0.34 ± 0.02 g·L^−1^. Thus, metabolic engineering of *C. glutamicum* for NMA production was achieved.

### 3.4. Fed-Batch Production of NMA in Bioreactors

**Figure 6:**
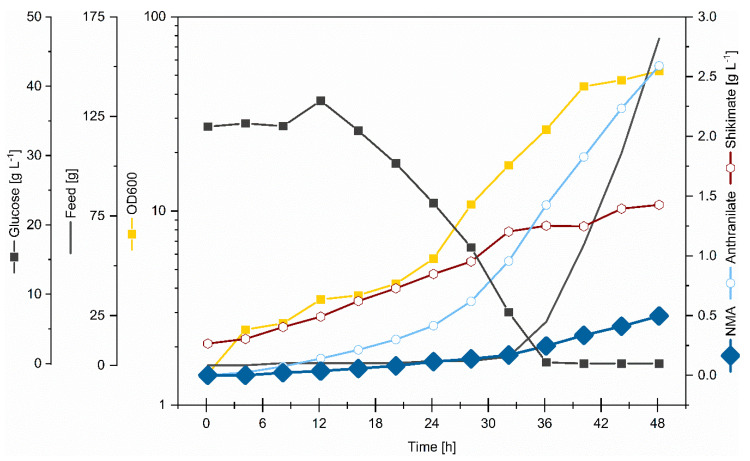
NMA production by *C. glutamicum* strains NMA105 in bioreactors operated in fed-batch mode. The cultivation (initial volume of 2 L) was performed in minimal medium supplemented with 40 g·L^-1^ glucose (dark grey line with squares). The feed (dark grey line) contained 400 g·L^−1^ glucose and 150 g·L^−1^ (NH_4_)_2_SO_4_. 1 mM IPTG was added for induction of gene expression during inoculation. OD_600_ (yellow) and concentrations of anthranilate (light blue), shikimate (maroon), and NMA (dark blue) in the culture broth are depicted. One of two representative fermentations is shown.

For industrial applications, a production in larger volumes is preferable, which runs under controlled conditions to obtain a constant production titer. The stability of the NMA production of the metabolically engineered strain NMA105 was investigated in a fed-batch cultivation. Starting with a working volume of 2 L CGXII minimal medium containing 40 g·L^−1^ glucose as carbon source, 160 mL feed (400 g·L^−1^ and 150 g·L^−1^ (NH_4_)_2_SO_4_) was added in a controlled manner depending on the rDOS (see Section 2.2). In total, 104 g glucose was consumed during 48 h fed-batch cultivation with no residual substrate concentrations detectable in the cultivation broth. The strain showed slow growth to OD_600_ 5 in the first 24 h. In the following phase, growth was faster (growth rate of 0.12 h^−1^, which was comparable to the growth rate observed in shaking flasks), and a maximal optical density of 53 was reached (Figure 6). High concentrations of by-products accumulated, i.e., 1.4 g·L^−1^ of the intermediate shikimate and 2.6 g·L^−1^ of the direct precursor anthranilate (Figure 6). Compared to production in shaking flasks (Figure 5), a reduced product yield on glucose (4.8 mg·g^−1^ as compared to 8.4 mg·g^−1^ in shaking flask) and a comparable volumetric productivity were observed, but NMA accumulated to an about 1.5-fold higher titer (0.5 g·L^−1^ as compared to 0.34 g·L^−1^). Taken together, the fed-batch fermentation with the newly constructed *C. glutamicum* strain NMA105 showed stable production of NMA in bioreactors at the 2 L scale (Figure 6). A final titer of 0.5 g·L^−1^ with a volumetric productivity of 0.01 g·L^−1^·h^−1^ and a yield of 4.8 mg·g^−1^ glucose was achieved.

## 4. Discussion

*N*-methylanthranilate production was achieved by applying the plant enzyme *N*-methylanthranilate transferase ANMT of *R. graveolens* in a newly metabolically engineered *C. glutamicum* anthranilate overproducer. *N*-methylanthranilate is known as precursor for several industrially and medically relevant compounds. ANMT of *R. graveolens* showed a narrow substrate specificity when various amino benzoic or benzoic acids or phenolic derivatives were tested as substrates [16]. However, feeding *O*-methylanthranilate (OMA) to *E. coli* expressing ANMT led to production of the flavoring compound *O*-methyl-*N*-methylanthranilate [15]. Hypothetically, ANMT could also be an interesting candidate to produce the pharmaceutically interesting compounds *O*-propyl- or *O*-isopropyl-*N*-methylanthranilate [22,23]. In the biosynthesis of acridone alkaloids, e.g., in *R. graveolens*, *N*-methylation of anthranilate catalyzed by ANMT is a key step preceding CoA activation and, thus, separating primary metabolism (here tryptophan synthesis) from secondary metabolism [16,19]. Recently, production of about 26 mg·L^−1^ 1,3-dihydroxy−10-methylacridone [65] and about 18 mg·L^−1^ 4-hydroxy-1-methyl-2(1H)-quinolone [66] were established in *E. coli* coexpressing *anmt* from *R. graveloens*, anthranilate coenzyme A ligase from *P. aeruginosa*, and acridone synthase of *R. graveolens* or the anthraniloyl-CoA anthraniloyltransferase from *P. aeruginosa*. In these biosynthesis pathways, one molecule of NMA is required per one molecule 1,3-dihydroxy-10-methylacridone or 4-hydroxy-1-methyl-2(1H)-quinolone [65,66]. The NMA-producing *C. glutamicum* strain NMA105 developed here may in the future be used in combination with this engineered *E. coli* strain, possibly as synthetic consortium [67,68], or *C. glutamicum* NMA105 itself may be engineered for production of acridone alkaloids.

Biosynthesis of *N*-alkylated amino acids can be catalyzed by other enzymes besides *N*-methyltransferases. However, while reductive amination using free ammonia is known for many enzymes, only few enzyme classes accept alkyl amines for *N*-alkylation, e.g., opine dehydrogenases, *N*-methyl amino acid dehydrogenases, ketimine reductases, pyrroline-5-carboxylate reductases, or imine reductases [12]. These processes differ regarding the substrate spectra of the involved enzymes. For example, anthranilate *N*-methylation described here as well as *N*-methylglutamate production established in *Pseudomonas putida* using *N*-methylglutamate synthase and γ-glutamylmethylamide synthetase of the methylamine assimilation pathway of *Methylobacterium extorquens* [13] have narrow substrate spectra (e.g., GMAS from *Methylovorus mays* also forms γ-glutamylethylamide, also known as theanine [69]) compared with *N*-alkylation using the imine reductase DpkA of *Pseudomonas putida* [12]. Several methylated or ethylated amino acids could be produced by *C. glutamicum* using the wild-type or a mutant version of DpkA and either MMA or ethylamine as substrates [14,34,35]. With respect to aromatic amino acids, *N*-methyl-l-phenylalanine could be obtained from phenylpyruvate by enzyme catalysis using DpkA and MMA [12]; however, production of NMA via DpkA by *N*-alkylamination of a carbonyl precursor of NMA has not been described.

The NMA process described here showed lower titers (0.5 g·L^−1^) than the processes depending on reductive alkylamination using MMA (about 32 g·L^−1^ *N*-methylalanine [34] and about 9 g·L^−1^ sarcosine [14]). This may be due to (a) higher activity of DpkA compared with ANMT, (b) better provision of the precursors pyruvate and glyoxalate than of anthranilate, and/or (c) the requirement of SAM for ANMT as compared to MMA for DpkA. Indeed, purified DpkA has a much higher activity (about 40 U·mg^−1^) [70] than purified ANMT (about 0.04 U·mg^−1^) [16]. Moreover, while ARO09(pEKEx3-*trpE*^FBR^) produced 3 g·L^−1^ anthranilate (Figure 3), the precursor strains used for production of *N*-methylalanine and sarcosine produced up to 45 g·L^−1^ pyruvate [71] and about 5 g·L^−1^ glycolate [72], respectively. Third, reductive methylamination using DpkA requires addition of MMA as methyl donor to the medium. This is beneficial since MMA has a low price, is readily available, is tolerated well by *C. glutamicum* [34], and because stoichiometric excess of MMA can be used to drive reductive *N*-methylation by mass action law.

Compared to NMA production by an engineered *E. coli* strain expressing the *N*-methyltransferase of *R. graveloens* [15], the NMA production by engineered *C. glutamicum* using the same enzyme described here resulted in about 12 times higher titers in shaking flask cultivation (370 mg·L^−1^ as compared to 29 mg·L^−1^). This may be due to the fact that, in this study, *C. glutamicum* was metabolically engineered for improved supply of the direct NMA precursor anthranilate as, e.g., strain ARO09(pEKEx3-*trpE*^FBR^) produced about 3 g·L^−1^ anthranilate. Moreover, while the *E. coli* relied on native SAM regeneration [15], in *C. glutamicum* the endogenous gene for SAM regeneration *sahH* was overexpressed to increase SAM regeneration, and NMA production was improved 1.36-fold (compare 0.34 ± 0.02 g·L^−1^ for NMA105 with 0.25 ± 0.02 g·L^−1^ for NMA104 in Figure 5). Two bottlenecks observed with the *C. glutamicum* strain engineered here may be overcome by future metabolic engineering: incomplete conversion of shikimate to anthranilate and incomplete *N*-methylation of anthranilate by SAM-dependent ANMT. To improve conversion of shikimate to anthranilate from about half to full conversion (compare about 1.4 g·L^−1^ of shikimate and 2.6 g·L^−1^ anthranilate produced by NMA105 in bioreactor cultivation; Figure 6), expression of the operon *aroCKB* encoding chorismate synthase, shikimate kinase, and 3-dehydroquinate synthase may be boosted, e.g., by changing the endogenous promoter for the strong promoter P*tuf* and using shikimate kinase from *Methanocaldococcus jannaschii* as shown previously [36]. In addition, various studies have shown that deletion of the chorismate mutase will increase the carbon flux towards tryptophan biosynthesis [36,40,73].

SAM-dependent *N*-methylation of anthranilate by ANMT from *R. graveloens* represents the second bottleneck. ANMT from *R. graveolens* shows high affinity for its substrates (K_M_ of 7.1 µM for anthranilate and K_M_ of 3.3 µM for SAM), and inhibition by its product NMA has not been described [16]. On the other hand, the inherently low activity of ANMT as compared, e.g., to DpkA (see above) may limit conversion of anthranilate to NMA. Importantly, regeneration of the methyl donor SAM (Figure 1A) is critical in all SAM-dependent methylation reactions. This is even more important for ANMT from *R. graveolens* because it is inhibited by SAH with a K_I_ value of 37.2 µM [74]. As shown here and elsewhere [41], overexpression of one gene of the SAM regeneration system (Figure 1A), *S*-adenosylhomocysteine (SAH) hydrolase gene *sahH*, partly overcame SAM limitation since conversion of anthranilate to NMA was improved 1.36-fold (Figure 5). This may be due to reduced inhibition of ANMT from *R. graveolens* by SAH (see above) and/or better SAM regeneration. Irrespective of *sahH* overexpression, not more than about 14 mol% of anthranilate was *N*-methylated to NMA (Figure 5). As shown for OMA production [41], overexpression of SAM synthetase gene *metK* in addition to *sahH* improved SAM regeneration, whereas deletion of cystathionin-γ-synthase gene *metB* and of *mcbR* and *cg3031* that code for transcriptional regulators involved in regulation of methionine biosynthesis were not beneficial. Addition of methionine even reduced the production [41]. These changes and abolishing pathways competing for SAM and its precursor by deletion of homoserine kinase gene *thrB* along with overexpression of *metK* and *vgb*, coding for methionine adenosyltransferase and *Vitreoscilla* hemoglobin, led to a *C. glutamicum* strain secreting about 0.2 g·L^−1^ SAM within 48 h [75]. In addition to improving SAM regeneration (as shown here by *sahH* overexpression), it may be beneficial for NMA production to increase SAM biosynthesis and, therefore, the intracellular concentration of SAM. Thus, possibly, NMA production may be improved by overexpression of SAM biosynthesis genes such as *metK,* or by de-repression of SAM biosynthesis, e.g., via deletion of *mcbR,* or by deletion of genes for enzymes competing with use of SAM or of SAM biosynthetic precursors such as *thrB*.

NMA may inhibit anthranilate biosynthesis since NMA was not produced in addition to anthranilate, while the combined titer of NMA and anthranilate remained similar when comparing strains NMA103, NMA104, and NMA105 (Figure 5). Enzymes that are inhibited by NMA have not been described to date. However, product inhibition of anthranilate synthase by anthranilate is known, e.g., in *Streptomyces* [76], which belongs to the actinobacteria as *C. glutamicum*, and in *Salmonella typhimurium* with a K_I_ of 0.06 mM anthranilate [77]. Here, we used the *E. coli* enzyme TrpE, which is known to be inhibited by tryptophan, which binds at a site distant from the active center (allosteric regulation) [78]. In the mutant TrpE^S40F^, Trp binding is lost as well as allosteric inhibition by Trp [78]. Product inhibition by anthranilate is expected to involve binding to the active center. Since NMA differs from anthranilate just by the *N*-methyl group, it is conceivable that NMA inhibits in a similar way as anthranilate. This may explain that upon NMA production the anthranilate titer decreased (Figure 5).

NMA also affected growth of *C. glutamicum* (34 mM or 5 g·L^−1^ reduced the growth rate to half-maximal; Figure 2), but to a lesser extent than OMA, for which a complete growth inhibition was observed at 2 g·L^−1^ OMA [41]. Inhibition of growth by OMA was overcome by application of a tributyrin-based extraction method [41]. This approach likely cannot be transferred directly to the NMA process since OMA contains a methylated carboxy group, whereas the amino group is methylated in NMA. Adaptive laboratory evolution (ALE) is an efficient method to select more tolerant strains and has been applied to *C. glutamicum* to select strains with improved tolerance to methanol [79,80,81] or lignocellulose-derived inhibitors [82].

Taken together, this study characterized NMA production by metabolically engineered *C. glutamicum,* and a first bioreactor process leading to a final titer of 0.5 g·L^−1^ NMA with a volumetric productivity of 0.01 g·L^−1^·h^−1^ and a yield of 4.8 mg·g^−1^ glucose was achieved. This strain provides the basis to develop an industrially competitive NMA process and shows potential to enable access to a fermentative route to pharmaceutically relevant secondary metabolites such as the acridone alkaloids.

## Figures and Tables

**Table 1:** Bacterial strains used in this study.

Strains	Description	Source
*Corynebacterium glutamicum*		
WT	*C. glutamicum* wild-type strain ATCC13032	ATCC
C1*	Genome-reduced chassis strain derived from	[42]
ARO01	Δ*vdh*::*P_ilvC_-aroG*^D146N^ mutant of C1*	This work
ARO02	Δ*ldhA* mutant of ARO01	This work
ARO03	Δ*sugR* mutant of ARO02	This work
ARO04	Δ*aroR*::*P_ilvC_-aroF* mutant of ARO03	This work
ARO05	Δ*qsuABCD*::*P_tuf_-qsuC* mutant of ARO04	This work
ARO06	Δ*ppc*::*P_sod_-aroB* mutant of ARO05	This work
ARO07	Δ*P_tkt_*::*P_tuf_-tkt* mutant of ARO06	This work
ARO08	Δ*iolR*::*P_tuf_-aroE* mutant of ARO07	This work
ARO09	Δ*sugR*::*sugR* mutant of ARO08	This work
NMA100	ARO09 carrying pEKEx3 and pGold	This work
NMA101	ARO09 carrying pEKEx3 and pGold-*anmt*	This work
NMA102	ARO09 carrying pEKEx3 and pGold-*anmt*-*sahH*	This work
NMA103	ARO09 carrying pEKEx3-*trpE*^FBR^ and pGold	This work
NMA104	ARO09 carrying pEKEx3-*trpE*^FBR^ and pGold-*anmt*	This work
NMA105	ARO09 carrying pEKEx3-*trpE*^FBR^ and pGold-*anmt*-*sahH*	This work
*Escherichia coli*		
S17-1	*recA pro hsdR* RP4-2-Tc::Mu-Km::Tn7	[45]
DH5α	*F-thi-1 endA1 hsdr17(r-, m-) supE44 1lacU169 (Φ* *80lacZ1M15) recA1 gyrA96*	[43]

**Table 2:** Oligonucleotides used in this study.

Name	Oligonucleotide Sequence (5′ to 3′)
vdh-conf-fw	GACCTCTAGGGCAGCAGTG
vdh-conf-rv	CTGTTCAGCGGATTAGCG
ldhA-conf-fw	TGATGGCACCAGTTGCGATGT
ldhA-conf-rv	CCATGATGCAGGATGGAGTA
sugR-conf-fw	CGAGATGCTGTGGTTTTGAG
sugR-conf-rv	GCTTATCGGGTGTGGGAATG
US-aroR-fw	CCTGCAGGTCGACTCTAGAGCGATGCAGAATAATGCAGTTAG
US-aroR-rv	CGGAGCTTGCCTGGGAGTTTGGAACCTTAACACACTTTC
PilvC-aroR-fw	GAAAGTGTGTTAAGGTTCCAAACTCCCAGGCAAGCTCCGCGC
PilvC-aroR-rv	**GAAAAAACCTCCTTTAGTGTGTAGTTAAGTT**ATGGTGATGGGAGAAAATCTCGCCTTTCG
DS-aroR-fw	AT**CACCATAACTTAACTACACACTAAAGGAGGTTTTTTC**ATGAGTTCTCCAGTCTCACTCGAAAAC
DS-aroR-rv	GAATTCGAGCTCGGTACCCGGGCAATGCGCAAGCCCTCTGGG
aroR-conf-fw	GGAACTCCCGTTGAGGTG
aroR-conf-rv	GTGGTACGAGCGCCGATTG
US-qsuA-fw	CCTGCAGGTCGACTCTAGAGGTTGGCAGCGCAACCAGTC
US-qsuA-rv	CTACTGACACGCTAAAACGCTGTCGATCCTGTTCATCG
Ptuf-qsuC-fw	CGATGAACAGGATCGACAGCGTTTTAGCGTGTCAGTAG
Ptuf-qsuC-rv	**CTGAAGGGCCTCCTTTC** TCCTCCTGGACTTCGTGG
qsuC-fw	GGA**GAAAGGAGGCCCTTCAG**ATGCCTGGAAAAATTCTCCTCC
qsuC-rv	GTCGAGGTTTTACTGACTCTTCTACTTTTTGAGATTTGCCAGG
DS-qsuD-fw	CTCAAAAAGTAGAAGAGTCAGTAAAACCTCGACGC
DS-qsuD-rv	GAATTCGAGCTCGGTACCCGGGATTTCGCGGATGGGTCTAAGTATG
qsu-conf-fw	GTTCGTGGACAAGTGTGGTGG
qsu-conf-rv	GTTCGTGGACAAGTGTGGTGG
US-ppc-fw	GCCTGCAGGTCGACTCTAGAGCGCTCAGGAAGTGTGCAAGGC
US-ppc-rv	GTACTACCCAGCCGGCTGGGGATCCCTACTTTAAACACTCTTTCACATTGAGGGTG
Psod-aroB-fw	AATGTGAAAGAGTGTTTAAAGTAGGAAGCGCCTCATCAGCGGTAAC
Psod-aroB-rv	**CTCCTTTAAAAATAAGTCGCCTACC** AAAATCCTTTCGTAGGTTTCCGC
aroB-fw	GCGGAAACCTACGAAAGGATTTT**GGTAGGCGACTTATTTTTAAAGGAGGTTTTTT**ATGAGCGCAGTGCAGATTTTC
aroB-rv	CTTCTCTCATCCGCCAAAATTAGTGGCTGATTGCCTCATAAG
Term-aroB-fw	CTTATGAGGCAATCAGCCACTAATTTTGGCGGATGAGAGAAG
Term-aroB-rv	AGTACTACCCAGCCGGCTGGGGATCCAAAAGAGTTTGTAGAAACGC
DS-ppc-fw	TGAAAGAGTGTTTAAAGTAGGGATCCCCAGCCGGCTGGGTAGTAC
DS-ppc-rv	GAATTCGAGCTCGGTACCCGGGCAGTGGGGAGACAACAGGTCG
ppc-conf-fw	CCGTCGGGAAACAGTTCCCC
ppc-conf-rv	GCAGACCCGTAAGTCCCTTGC
US-tkt-fw	GCATGCCTGCAGGTCGACTCTAGAGTGACCCAGGTGGACGCCAAC
US-tkt-rv	GTGGACATTCGCAGGGTAACGGCCAAGGTGTGATCAATCTTAAGTC
Ptuf-tkt-fw	GACTTAAGATTGATCACACCTTGGCCGTTACCCTGCGAATGTCCAC
Ptuf-tkt-rv	CGTCAAGGTGGTCATCTGAAGGGCCTCCTTTCTGTATGTCCTCCTGGACTTC
DS-tkt-fw	CAGGAGGACATACA**GAAAGGAGGCCCTTCAG**ATGACCACCTTGACGCTGTC
DS-tkt-rv	GAATTCGAGCTCGGTACCCGGGTGGCGGTACTCAGGGTGTCC
tkt-conf-fw	GTTCCCGAATCAATCTTTTTAATG
tkt-conf-rv	GACCCTGGCCAAGAGGGCCAGTG
US-iolR-fw	GCCTGCAGGTCGACTCTAGAGCGACCCTCACGATCGCATG
US-iolR-rv	CTACTGACACGCTAAAACGCGATGTCTCCTTTCGTTGCCC
Ptuf-aroE-fw	GGGCAACGAAAGGAGACATCGCGTTTTAGCGTGTCAGTAG
Ptuf-aroE-rv	CCCA**TCTGAAGGGCCTCCTTTC**TCCTCCTGGACTTCGTGGTG
aroE-fw	GGA**GAAAGGAGGCCCTTCAG**ATGGGTTCTCACATCACTCACCG
aroE-rv	CAGAAGGGCTCTTTGGTTTATTTCTTAGTGTTCTTCTGAGATGCCTAAAGACTC
DS-iolR-fw	GAGTCTTTAGGCATCTCAGAAGAACACTAAGAAATAAACCAAAGAGCCCTTCTG
DS-iolR-rv	GAATTCGAGCTCGGTACCCGGGCGCTCTCCATCCGCTGGAC
iolR-conf-fw	CAGATAGAGGAACCCAAGGCG
iolR-conf-rv	GGACTTCGTGAGTGCTCGTC
sugR_reintegr-fw	CTGCAGGTCGACTCTAGAGCCTGCGCAGGGACCCTAATAAG
sugR_reintegr-rv	GAATTCGAGCTCGGTACCCGGGCCTGCAGTAAAAGATTCCCGC
x3-trpE-fw	CCTGCAGGTCGACTCTAGAG**GAAAGGAGGCCCTTCAG**ATGCAAACACAAAAACCGACTCTCGAACTG
x3-trpE-rv	AAAACGACGGCCAGTGAATTTCAGAAAGTCTCCTGTGCATGATGCGC
pGANMT-sahH-fw	ATGAGCTCGGTACC**CGGGCGGGACGAAGAGAACCGTTACAAGAATAAAGGAGGTTTTTT**ATGGCACAGGTTATGGACTTC
pGANMT-sahH-rv	CTGCAGGTCGACTCTAGAGTTAGTAGCGGTAGTGCTCCGG

Ribosomal binding sites are in bold, and binding regions of Gibson oligonucleotides are underlined.

**Table 3:** List of plasmids used in this study.

Plasmids	Description	Source
pK19*mobsacB*	Km^R^; *E. coli*/*C. glutamicum* shuttle vector for construction of insertion and deletion mutants in *C. glutamicum* (pK19 *oriV_Ec_ sacB lacZα*)	[50]
pK19-Δ*vdh*::*P_ilvC_-aroG*^D146N^	pK19*mobsacB* with a construct for replacement of *vdh* (cg2953) by *aroG*^D146N^ from *E. coli* under control of *C. glutamicum* promoter *P_ilvC_*	[36]
pK19-Δ*ldhA*	pK19*mobsacB* with a construct for deletion of *ldhA* (cg3219)	[52]
pK19-Δ*sugR*	pK19*mobsacB* with a construct for deletion of *sugR* (cg2115)	[53]
pK19-Δ*aroR*::*P_ilvC_*	pK19*mobsacB* with a construct for replacement of *aroR* and the native promoter of *aroF* by *C. glutamicum* promoter *P_ilvC_* and an artificial RBS	This work
pK19-Δ*qsuABCD*::*P_tuf_-qsuC*	pK19*mobsacB* with a construct for replacement of *qsuABCD* (cg0501-cg0504) by *qsuC* (cg0503) with an artificial RBS under control of *C. glutamicum* promoter *P_tuf_*	This work
pK19-Δ*ppc*::*P_sod_-aroB*	pK19*mobsacB* with a construct for replacement of *ppc* (cg1787) by *aroB* (cg1827) with an artificial RBS under control of *C. glutamicum* promoter *P_sod_*	This work
pK19-Δ*P_tkt_*::*P_tuf_*	pK19*mobsacB* with a construct for replacement of the *tkt* (cg1774) promoter by *C. glutamicum* promoter *P_tuf_* and artificial RBS	This work
pK19-Δ*iolR*::*P_tuf_-aroE*	pK19*mobsacB* with a construct for replacement of *iolR* (cg0196) by *aroE* (cg1835) with an artificial RBS under control of *C. glutamicum* promoter *P_tuf_*	This work
pK19-Δ*sugR*::*sugR*	pK19*mobsacB* with a construct for reintegration of *sugR* (cg2115) into its native locus	This work
pEKEx3	Spec^R^, *P_tac_lacI^q^*, pBL1 *oriV_Cg_*, *C. glutamicum*/*E. coli* expression shuttle vector	[54]
pEKEx3-*trpE*^FBR^	Spec^R^, pEKEx3 overexpressing *trpE*^S40F^ from *E. coli* K12 containing an artificial RBS	This work
pEC-XK99E	Km^R^, P*_trc_lacI^q^*, pGA1 *oriV_Ec_, C. glutamicum*/*E. coli* expression shuttle vector	[55]
pGold	Km^R^, P*_trc_lacI^q^*, pGA1 *oriV_Ec_, C. glutamicum*/*E. coli* expression shuttle vector with *Bsa*I recognition site for Golden Gate assembly	This work
pGold-*anmt*	Km^R^, pGold overexpressing codon harmonized *anmt* from *Ruta graveolens* with an artificial RBS	This work
pGold-*anmt*-*sahH*	Km^R^, pGold overexpressing a synthetic operon with codon harmonized *anmt* from *R. graveolens* with an artificial RBS and *sahH* from *C. glutamicum* with an artificial RBS	This work

## Supplementary Materials

The following figures are available online at https://www.mdpi.com/2076-2607/8/6/866/s1, Figure S1: Carbon flux analysis of anthranilate producing *C. glutamicum* ARO strains, Figure S2: Carbon flux analysis of *C. glutamicum* ARO strains harboring the plasmid pEKEx3, Table S1: Codon harmonized nucleotide sequence (5′-3′) of the plant gene *anmt*.
