# Supplementary material for: Fermentative N-Methylanthranilate Production by Engineered Corynebacterium glutamicum
Source: Microorganisms. 2020 Jun 8;8(6):866. doi: 10.3390/microorganisms8060866 (PMC7356990; doi:10.3390/microorganisms8060866)
Supplement: Supplementary file 1 [file microorganisms-08-00866-s001.zip › Supplementary Files/Figure S1.pdf]

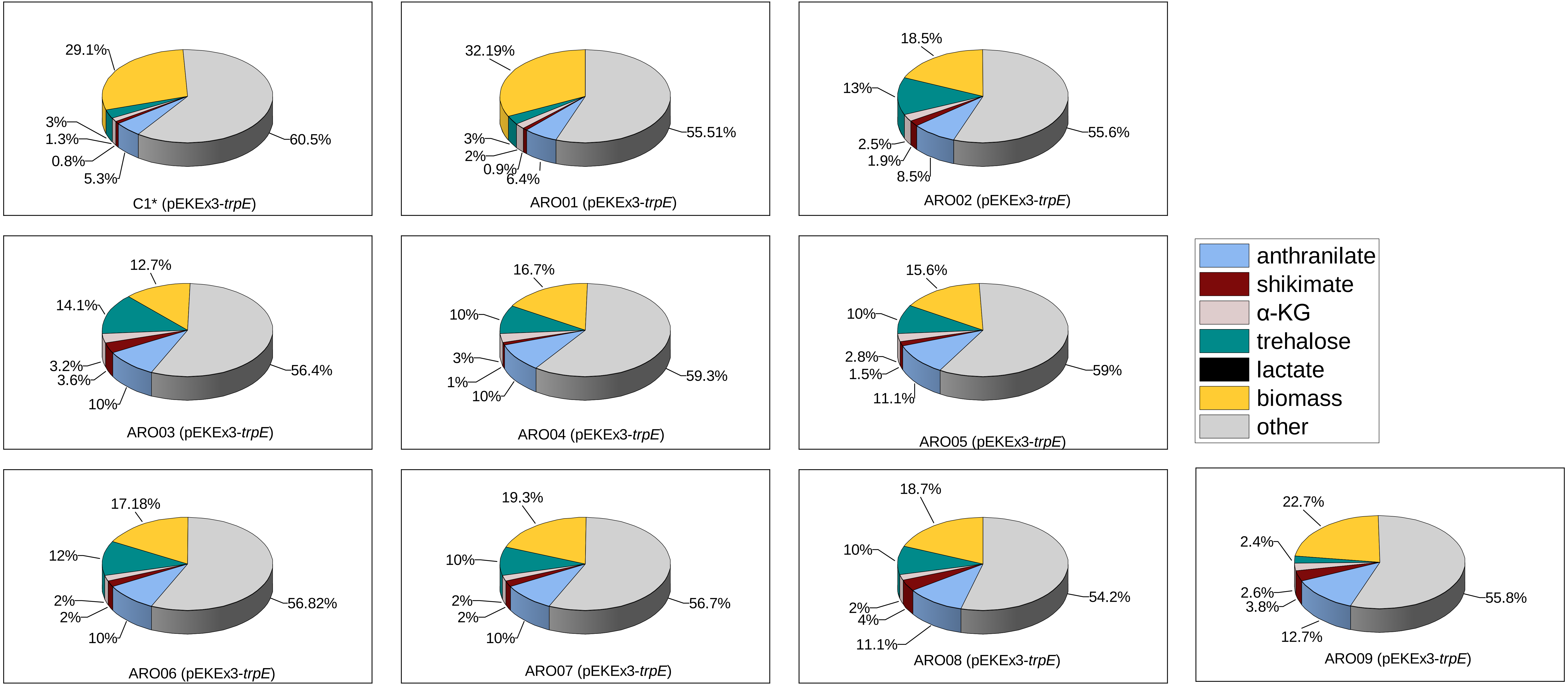
29.1%
3%
1.3%
0.8%
5.3%
60.5%
C1* (pEKEx3-trpE)
32.19%
3%
2%
0.9%
6.4%
55.51%
ARO01 (pEKEx3-trpE)
18.5%
13%
2.5%
1.9%
8.5%
55.6%
ARO02 (pEKEx3-trpE)
12.7%
14.1%
3.2%
3.6%
10%
56.4%
ARO03 (pEKEx3-trpE)
16.7%
10%
3%
1%
10%
59.3%
ARO04 (pEKEx3-trpE)
15.6%
10%
2.8%
1.5%
11.1%
59%
ARO05 (pEKEx3-trpE)
anthranilate
shikimate
α-KG
trehalose
lactate
biomass
other
17.18%
12%
2%
2%
10%
56.82%
ARO06 (pEKEx3-trpE)
19.3%
10%
2%
2%
10%
56.7%
ARO07 (pEKEx3-trpE)
18.7%
10%
2%
4%
11.1%
54.2%
ARO08 (pEKEx3-trpE)
22.7%
2.4%
2.6%
3.8%
12.7%
55.8%
ARO09 (pEKEx3-trpE)
