# Supplementary figures and images for: Fermentative N-Methylanthranilate Production by Engineered Corynebacterium glutamicum
Source: Microorganisms. 2020 Jun 8;8(6):866. doi: 10.3390/microorganisms8060866 (PMC7356990; doi:10.3390/microorganisms8060866)

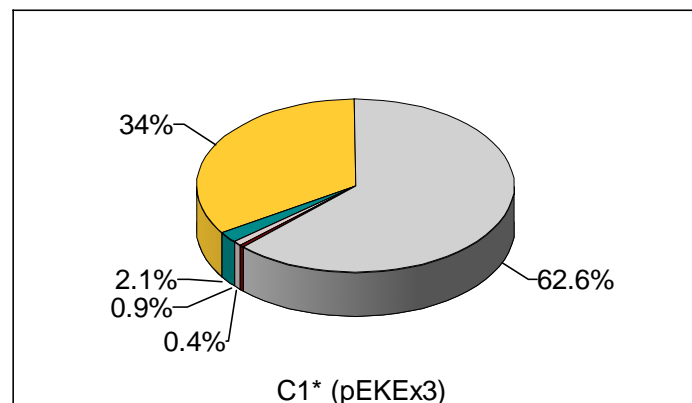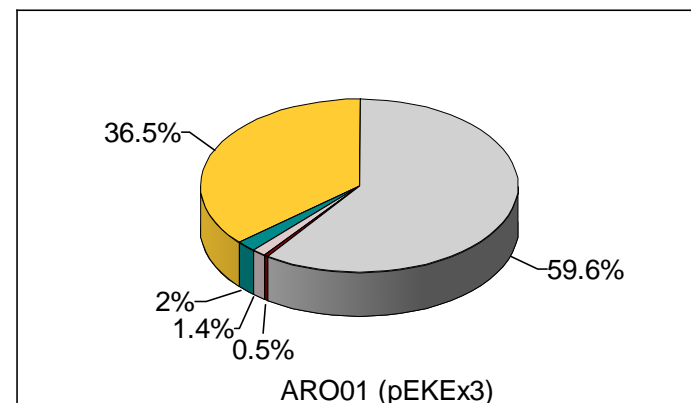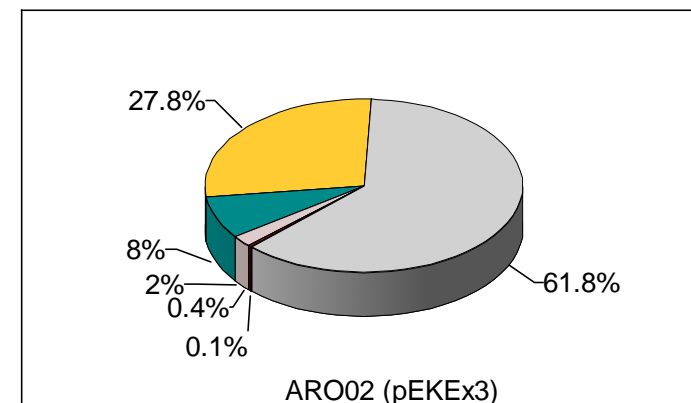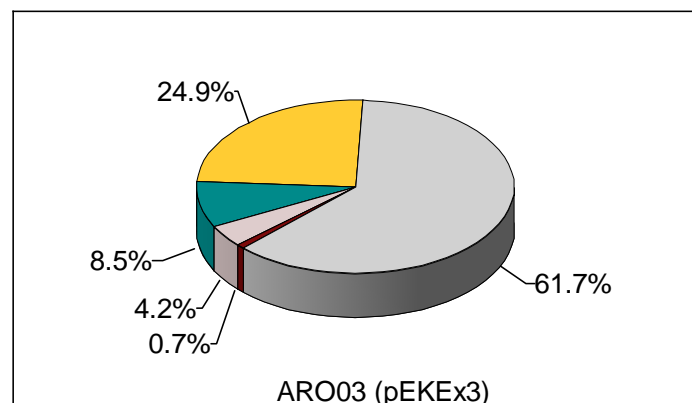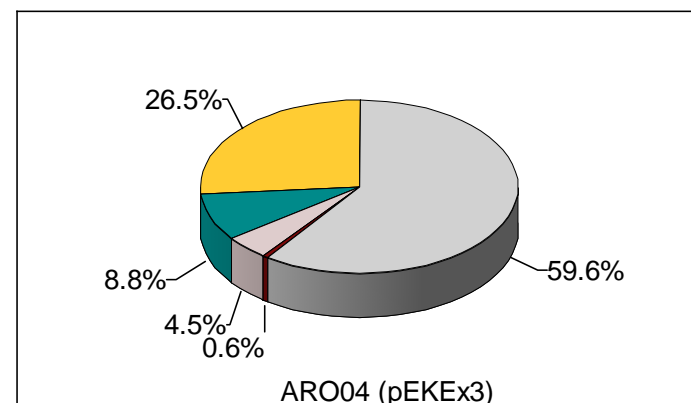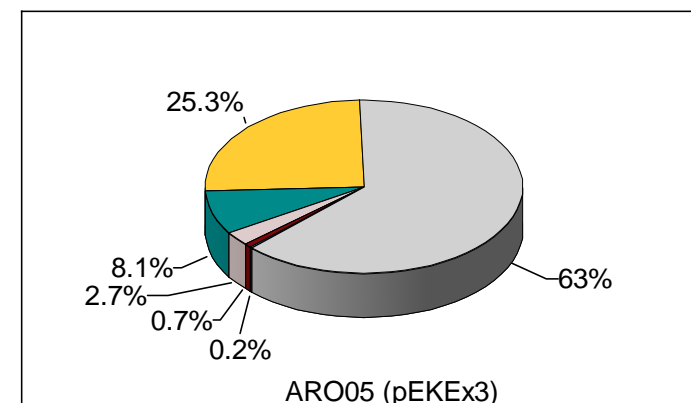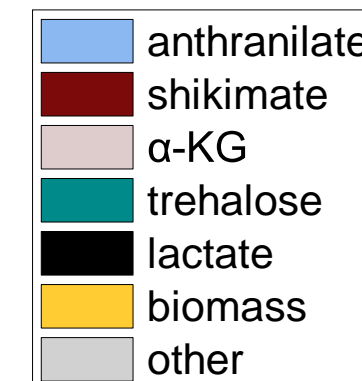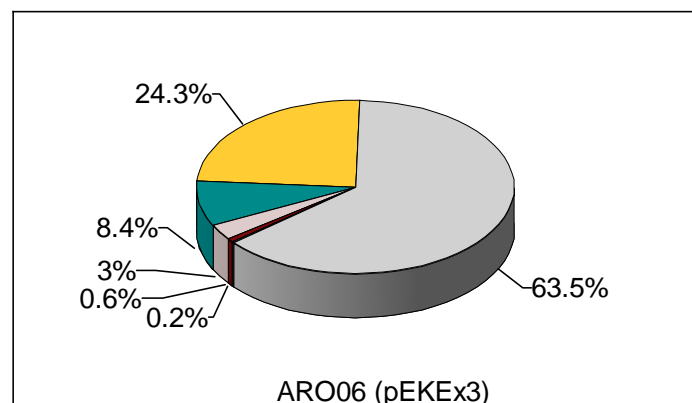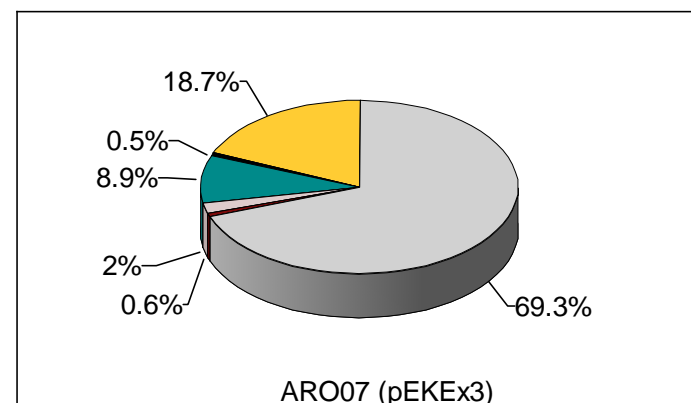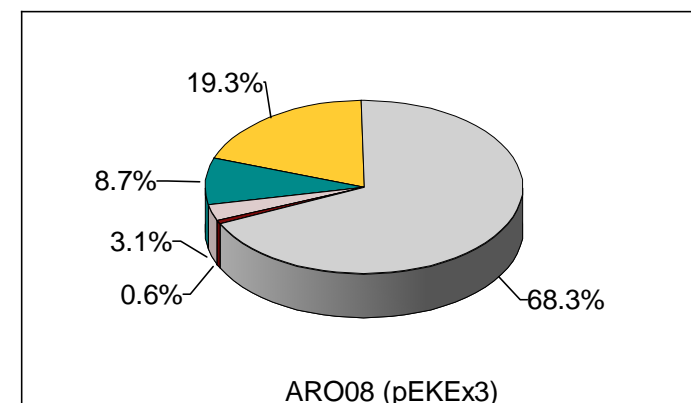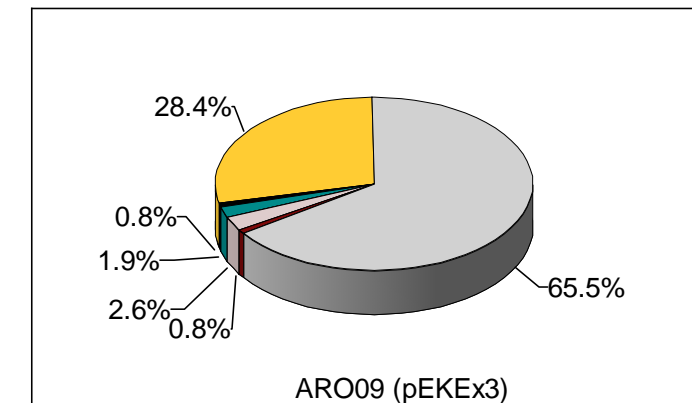

Supplement: Supplementary file 1 [file microorganisms-08-00866-s001.zip › Supplementary Files/Figure S2.pdf]
